# Limitations in the Diagnosis of Noncompaction Cardiomyopathy by Echocardiography

**DOI:** 10.5935/abc.20170152

**Published:** 2017-11

**Authors:** Viviane Tiemi Hotta, Sabrina Cunha Tendolo, Ana Clara Tude Rodrigues, Fábio Fernandes, Luciano Nastari, Charles Mady

**Affiliations:** 1 Instituto do Coração (InCor) do Hospital das Clínicas da Faculdade de Medicina da Universidade de São Paulo (HCFMUSP), São Paulo, SP - Brazil; 2 Fleury Medicina e Saúde, São Paulo, SP - Brazil; 3 Hospital das Clínicas - FMUSP, São Paulo, SP - Brazil; 4 Hospital Israelita Albert Einstein, São Paulo, SP - Brazil

**Keywords:** Cardiomyopathies, Heart Failure, Diagnostic Imaging / trends, Echocardiography

Noncompacted myocardium (NCM) was first reported by Grant in 1926 as a heterogeneous myocardial disorder characterized by prominent ventricular trabeculation, intratrabecular recesses, and bilayered myocardium composed by a compacted and a noncompacted layer.^1,2^ It can occur isolated or associated with other cardiomyopathies, complex syndromes, metabolic disorders and congenital heart diseases, such as Ebstein's anomaly, left ventricular (LV) or right ventricular outflow tract obstruction, bicuspid aortic valve, cyanotic congenital heart diseases, and coronary artery anomalies. Although NCM usually affects the left ventricle, it can also affect both ventricles or the right ventricle alone.^3^

The etiology of LV noncompaction is uncertain, and several etiological bases have been implicated. It is believed to be due to pathogenic mechanisms resulting in a failure in the final phase of myocardial morphogenesis, or myocardial compaction. Increasing evidence has supported a genetic base by identifying mutation in the genes that encode sarcomeric, cytoskeletal and nuclear membrane proteins.^4-6^

Although considered rare by some authors, NCM incidence and prevalence are uncertain. Ritter et al.^7^ have reported a 0.05% prevalence in all echocardiographic exams of a large institution. Patients with heart failure (HF) have been reported to have a 4% prevalence of NCM.^8^

Currently, it is controversial whether NCM is a distinct cardiomyopathy or a morphological characteristic shared by different heart diseases. Thus, while the World Health Organization/International Society and Federation of Cardiology considers NCM an unclassified cardiomyopathy, the American Heart Association considers it a primary genetic cardiomyopathy.^9,10^ The most recent classification of cardiomyopathies proposed by the European Society of Cardiology Working Group on Myocardial and Pericardial Diseases considers NCM an unclassified familial cardiomyopathy.^11^

The clinical presentation can occur at any age, being highly variable. Patients can be asymptomatic or have symptoms of severe HF, associated or not with lethal arrhythmias, sudden cardiac death and thromboembolic events.^12^

Many asymptomatic patients are identified incidentally by an echocardiography performed for assessment of cardiac murmur or for familial screening after identifying an index case.^12^

Symptoms of HF occur in more than half of the patients with NCM, LV dysfunction being reported in up to 84% of them. In addition, arrhythmias are common: atrial fibrillation can affect 25% of adult patients, and ventricular tachyarrhythmias, up to 47% of patients. The occurrence of thromboembolic events, such as stroke, transient ischemic attack, pulmonary embolism and mesenteric ischemia, ranges from 0 to 38%, according to studies published.^3,13,14^

Electrocardiographic abnormalities can be present in up to 90% of patients, being, however, unspecific. The most common findings include intraventricular conduction delay, LV hypertrophy, ventricular repolarization changes, and Wolff-Parkinson-White syndrome.^3,13,14^

The increasing advancement in imaging techniques, in addition to the increasing application of genetic tests for the diagnosis of NCM, significantly impacts on the understanding of the mechanisms involved in the NCM genesis and its clinical treatment.

Of the cardiac imaging techniques, echocardiography and cardiac magnetic resonance imaging (CMR) are the major diagnostic tools. Because of its large availability and easy access, in addition to no need for contrast agents, no radiation exposure, and mainly its low cost as compared to CMR, echocardiography is the first choice and most commonly used method for the diagnosis of NCM.^8,12-14^

Usually the diagnosis of NCM should be considered in the presence of a bilayered myocardium composed by one thinner epicardial layer and one thick endocardial layer with prominent trabeculations and deep intraventricular recesses. The trabeculations are mainly identified on two-dimensional (2D) mode, but can be evidenced on one-dimensional or M mode. Color Doppler imaging shows blood flow in those recesses in continuity with the left ventricle.^8,12-15^

Different echocardiographic criteria have been used to diagnose NCM, and the main ones used in clinical practice are as follows (Table 1):

**Table 1:** Criteria proposed for the diagnosis of noncompacted myocardium.^6,13^

Criterion	Chin et al.^2^	Jenni et al.^16^	Stöllberger et al.^17^
Number of patients	8	7	104
Phase of the cardiac cycle	End-diastole	End-systole	Trabeculations assessed at end-diastole; NCM and CM assessed at end-systole
View	Short axis parasternal views and/or apical views	Short axis parasternal	Conventional and modified views
NCM/CM ratio	-	>2	-

1. Jenni et al.^16^ consider for the diagnosis of NCM the presence of two myocardial layers, a thin compacted one (compacted myocardium - CM), and another thicker, noncompacted layer (NCM), with deep endomyocardial recesses filled with blood flow on color Doppler, in the absence of other cardiac abnormalities. A NCM/CM ratio > 2 is considered diagnostic. The measures should be acquired at end-systole on short axis parasternal view^16^ (Figure 1).
  **Figure 1:**
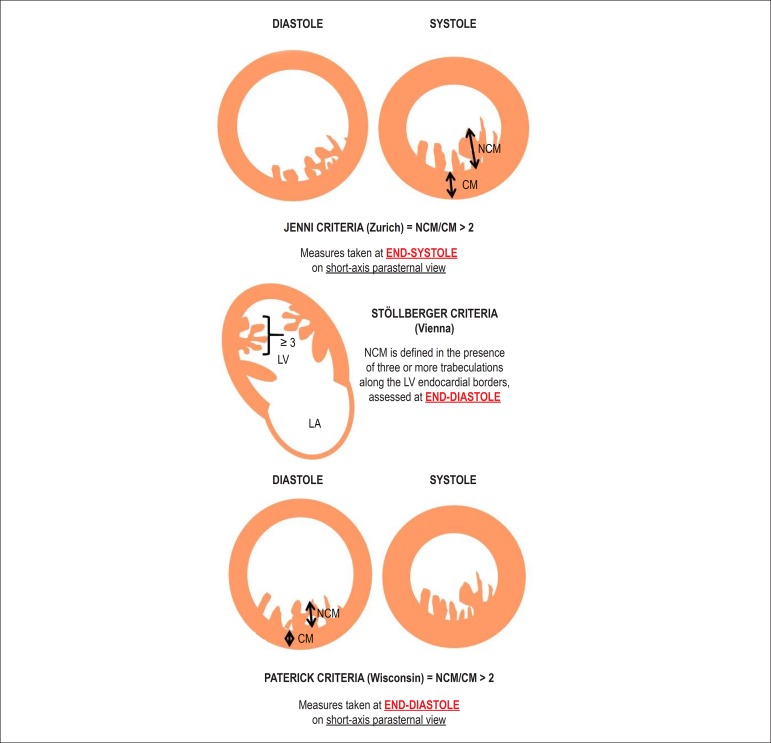
Criteria proposed for the diagnosis of noncompacted myocardium. NCM: noncompacted myocardium; CM: compacted myocardium; LV: left ventricle; LA: left atrium.
2. Chin et al.^2^ define NCM as the presence of excessively prominent ventricular trabeculations and progressively increased total thickness of the myocardial wall from the mitral valve and towards the apical region, characterized by CM/(NCM + CM) ≤ 0.5, assessed at end-diastole on short-axis parasternal views and/or apical views^17^ (Figure 2).
  **Figure 2:**
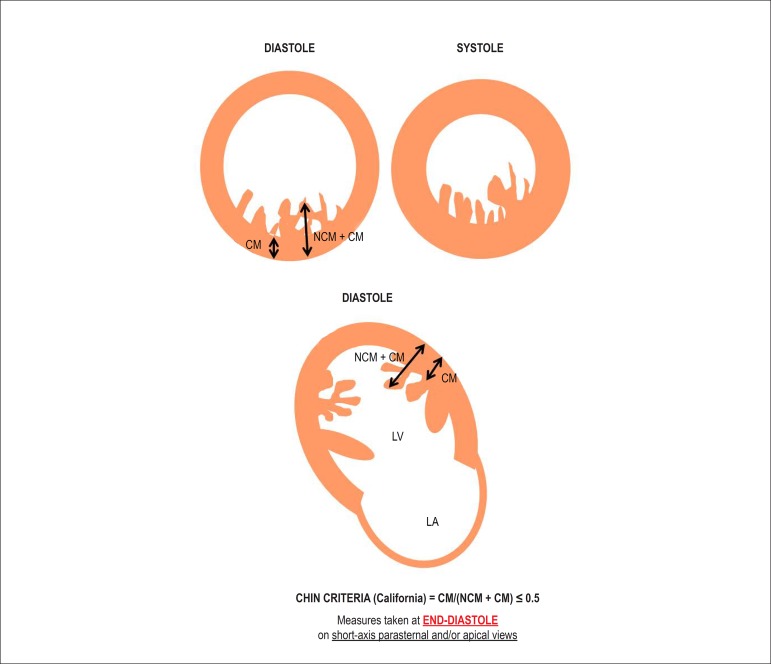
Criteria proposed by Chin for the diagnosis of noncompacted myocardium. NCM: noncompacted myocardium; CM: compacted myocardium; LV: left ventricle; LA: left atrium.
3. Stöllberger et al.^17^ define NCM as the presence of three or more trabeculations along the LV endocardial borders, different from the papillary muscles, false tendons and aberrant muscle bands, which move synchronized with the CM. In that study, the trabeculations were better visualized at end-diastole, while the bilayered myocardium was better assessed at end-systole^18^ (Figure 1).
4. Recently, Paterick et al.^13^ (Wisconsin) have proposed the diagnosis of NCM as a ratio NCM/CM > 2, with measures taken at end-diastole on short-axis parasternal view. This criterion requires clinical validation^13^ (Figure 1).

## Critical analysis

The diagnostic criteria described by Jenni et al.^16^ and Chin et al.^2^ are based on the measurement of NCM and CM thicknesses. However, the criteria differ regarding the cardiac cycle point at which the measurements should be taken. Chin et al.^2^ propose the measurements of NCM and CM thickness be performed at end-diastole, while Jenni et al. propose them at end-systole.^16^

The criteria proposed by Jenni et al.^16^ used an NCM/CM ratio > 2 at end-systole, generating higher specificity and lower sensitivity as compared to the criteria by Chin et al.,^2^ who use the CM/(NCM + CM) ratio ≤ 0.5. However, the increase in sensitivity is due to a decrease in specificity, as compared to the criteria by Jenni et al.^16^

A recent study has assessed the accuracy of the echocardiographic criteria described by Chin et al.,^2^ Jenni et al.^16^ and Stöllberger et al.^17^ for the diagnosis of NCM in patients with HF as compared to a control group of normal individuals. The size and the number of the trabeculations identified on apical view at end-diastole were assessed, as were the measurements of the NCM layer thickness on short-axis parasternal view at end-systole. Only concordant cases assessed by two reviewers were considered positive.^18^

In that study, the percentages of patients meeting the diagnostic criteria for NCM were as follows: Chin et al.^2^ criteria, 79%; Jenni et al.^16^ criteria, 64%; and Stöllberger et al.^17^ criteria, 53%. In that study, the Chin et al. criteria had higher sensitivity, however with a higher percentage of false-positive diagnoses. The correlation between the three echocardiographic criteria applied was weak, with only 30% of patients meeting all three criteria. All individuals of the control group had preserved ventricular dimensions and systolic function. Five control group individuals (4 black and 1 white) met at least one criterion for the diagnosis of NCM. This result emphasizes the limitation of the echocardiographic criteria to diagnose NCM, particularly in black individuals, leading to an excessive diagnosis of NCM.^18^ In that study, if the control group was formed by individuals with HF, those results might have been even more discrepant.

The criterion proposed by Paterick et al.^13^ showed good correlation with the CMR findings, and, according to those authors, that criterion provided more accurate measurements of NCM and CM layer thickness. However, those criteria have not been validated, requiring additional confirmation and comparison with other populations with cardiac structural disease before they are adopted as a feasible diagnostic option.^13^

Despite the increasingly frequent diagnosis of NCM, the echocardiographic criteria applied for that purpose are based on studies with limited numbers of patients and different methodologies. The point of the cardiac cycle at which the measurements of NCM and CM thickness are taken influences directly the relationship between the two layers assessed. Myocardial thickness is maximal at systole, and minimal at diastole, which directly affects the ratio between NCM and CM. In addition, the echocardiographic view on which those measurements are taken should be considered. Most criteria suggest that the measurements be taken on short-axis parasternal view; however, in daily clinical practice, measurements are more often taken on apical 4- and 2-chamber views. Finally, there is no consensus on the ratio between NCM and CM to be adopted as the diagnostic criterion, because of the lack of uniformity accepted for diagnosis.

In addition, some studies have shown a considerable number of young athletes meeting the NCM diagnostic criteria, emphasizing the lack of specificity of the current diagnostic criteria when applied to highly-trained athletes.^19^

Although infrequent, NCM has been reported in the right ventricle. However, in such cases, the diagnostic criteria are even more restricted as compared to those applied to the left ventricle on echocardiography, because of the limitation of the right ventricle echocardiographic analysis due to its complex geometry, which cannot be contemplated on only one echocardiographic view.^20,21^

Thus, despite the increasing knowledge on NCM by echocardiography professionals, the diagnostic bases of the criteria applied are frail. Therefore, studies with a larger number of patients diagnosed with NCM are required, in addition to uniformization of the views used for the measurements, and the identification of the most suitable point in the cardiac cycle for that purpose. Such studies should ideally compare healthy individuals and patients with HF, because some "normal" patients can meet the echocardiographic criteria for the diagnosis of NCM, with no apparent clinical finding and benign prognosis, considering that the prevalence of NCM seems higher in patients with HF. In addition, the definition of the diagnostic criteria should take into consideration the particularities of specific populations, such as highly-trained athletes and black individuals, as well as the right ventricular morphological characteristics.

## Conclusions

Echocardiography is the first choice and most commonly used cardiac imaging method for the diagnosis of NCM. Higher knowledge and understanding of NCM is the first step to increase diagnostic accuracy in echocardiography laboratories. However, the echocardiographic criteria used so far for that purpose are highly varied and have been based on studies with a reduced number of patients.

Advanced techniques, such as three-dimensional echocardiography, use of contrast agents to better define the endocardial borders, mainly in the apical region of patients with limited acoustic window, as well the analysis of myocardial strain by use of speckle tracking, are promising methods, with potential to increase the diagnostic accuracy of echocardiography in patients with NCM. The use of such techniques in clinical practice has increased in past years; however, the improvement of imaging methods requires study and constant redefinition of the echocardiographic criteria for the diagnosis of NCM.^22-25^

The high prevalence of NCM in low-risk populations, such as athletes and normal black individuals, suggests that the increase in LV trabeculations and recesses can represent a pattern of response to the chronic increment of preload. Thus, because of the current limitations for the diagnosis of NCM, integration of clinical and electrocardiographic assessments, as well as a multimodality approach with echocardiography and CMR, is suggested.^26-28^

In addition to the multimodality imaging approach, future perspectives include changes that suit different ethnicities and functional assessment based on multicenter and international collaboration, incorporating genetic data for a more accurate diagnosis of NCM.^26-28^
